# DUALS AND ASSISTED LIVING: EXAMINING ACCESS AND OUTCOMES

**DOI:** 10.1093/geroni/igac059.1651

**Published:** 2022-12-20

**Authors:** Kali Thomas, Hyunjee Kim

## Abstract

Assisted living, a popular long-term care option for older adults needing personal care assistance, is increasingly serving a vulnerable population of low-income older adults dually-enrolled in Medicare and Medicaid (duals). However, we know very little about the needs of this population, their access to assisted living, the quality of care duals are receiving, and how this varies across states and assisted living providers. This symposium will include five presentations using national data and focused on access to assisted living for duals and the quality of care that duals in assisted living settings receive. The first two presentations take different approaches to investigate access to assisted living and how it relates to Medicaid policy: one examines Medicaid policies and their relationship to geographic access, and the other presents results from an analysis examining the association between Medicaid financing and segregation of duals. The next three presentations highlight important findings related to the quality of care for duals in assisted living: one focuses on concentration of duals in assisted living and its relationship with hospitalization and nursing home placement, one examines injury-related emergency department visits among duals, and the final presentation discusses the ability of duals in assisted living to age in place toward the end of life. The discussant, an expert in Medicaid policy, will conclude with a discussion of states’ different approaches for covering services in assisted living through their Medicaid programs, highlighting the opportunities for increasing equitable access and ensuring high quality care is delivered to this vulnerable population.

